# A Study of the Effect of Pre-emptive Oral Clonidine on Intraoperative Haemodynamics and Surgical Field Quality During Functional Endoscopic Sinus Surgery Under General Anaesthesia

**DOI:** 10.7759/cureus.37918

**Published:** 2023-04-21

**Authors:** Kapil Khandelwal, Jayashree Sen

**Affiliations:** 1 Department of Anaesthesia, Jawaharlal Nehru Medical College (Datta Meghe Institute of Higher Education & Research), Wardha, IND

**Keywords:** functional endoscopic sinus surgery, surgical field quality, controlled hypotension, hypotensive anaesthesia, clonidine

## Abstract

Introduction

For endoscopic sinus surgery for rhinosinusitis, pre-emptive Alpha 2 agonist clonidine has been used to reduce sympathetic output, which lowers blood pressure and consequently surgical bleeding. The aim of this study was to analyze the effects of oral clonidine premedication in patients undergoing functional endoscopic sinus surgery.

Methods

This study was performed between December 2020 to November 2022 among two groups of 30 patients each: clonidine (200 μgm oral) and placebo. Parameters were recorded at baseline, 60 mins after administering the drugs, at induction, and at minutes five, 10, 20, 30, 45, 60, 75, 105, and 120. Six-point average category scale for bleeding was studied. IBM SPSS Statistics for Windows, Version 20.0 (Released 2011; IBM Corp., Armonk, New York, United States) was used for statistical analysis, and p < 0.05 was considered significant.

Results

Demographic criteria were statistically non-significant. Heart rate (HR) and mean arterial pressure (MAP) were statistically non-significant at baseline and the 120th min mark, and were significant at other time intervals. The blood loss grading was less in the clonidine group, and the difference was found to be statistically significant (P < 0.001).

Conclusion

Pre-emptive oral clonidine 200 mcg 60 min prior to induction was found to reduce surgical bleeding by controlling haemodynamics.

## Introduction

Functional endoscopic sinus surgery (FESS) has emerged as the gold standard for surgically treating paranasal sinus diseases [1]. The nasal mucosa has a rich blood supply, so, during surgery, poor visibility of local anatomy and critical structures results from heavy bleeding. This causes more tissue damage, extends the surgical time, develops postoperative adhesions, and determines whether the surgery is successful or not. A few of the factors that can influence the surgical field include the patient's physical health, co-occurring diseases like bleeding disorders, and/or any pre-existing vascular network conditions. Endoscopic sinus surgery can be performed while anesthesia is administered using a variety of methods, such as local anesthesia [2], vasoconstrictors like epinephrine, cocaine, and phenylephrine [2-4], or general anesthesia with regulated hypotension [5]. To create a "bloodless field" by producing controlled hypotension with certain drugs, viz: ꞵ blockers, nitroglycerine, sodium nitroprusside propofol, and inhaled anesthetics. For many years, clonidine, an α2-agonist, has been utilized as an antihypertensive. In this study, clonidine has been administered as a premedicant to induce hypotension during general anesthesia.

## Materials and methods

Study design

A prospective randomized double-blinded placebo-controlled study.

Study population

The patients, selected for FESS from the otorhinolaryngology outpatient department and were fulfilling the eligibility criteria for our study, were included.

Eligibility criteria

Inclusion Criteria

20-50 years age group; weight between 40-65 kg; either gender, the physical status I and II of American Society of Anesthesiology (ASA); patients selected for FESS.

Exclusion Criteria

Refusal to give consent; history of cardiovascular, pulmonary, renal, hepatic, endocrinal, or neurological disease; history of preoperative hypotension or obesity; anticipated difficult airway as per look, evaluate the 3-3-2 rule, Mallampati score, obstruction, and neck mobility (LEMON) criteria; cardiovascular system dysfunction as sinus bradycardia, conduction defects, heart blocks; patients taking antipsychotic drugs; patients on drugs as calcium channel blockers, digitalis, β-blockers; allergy to study drugs; currently pregnant or lactating patients.

This prospective, randomized, double-blinded, placebo-controlled study was conducted at Acharya Vinoba Bhave Rural Hospital (AVBRH), Jawaharlal Nehru Medical College (JNMC), Datta Meghe Institute of Higher Education & Research (DMIHER), Sawangi (Meghe), Wardha from December 2020 to November 2022, having taken informed written consent after proper counseling from each patient and the approval of the ethical committee of the institution. Prior permission from the head of the otorhinolaryngology department was sought for the study. The 60 patients that were selected for FESS were randomly allocated into groups: Group C (clonidine, n=30) and Group P (placebo, n=30).

Methods

Every patient received a thorough physical evaluation, biochemistry inquiry reports, an electrocardiogram, and a chest X-ray before going under anesthesia. The day before the operation, patients were given a tablet of alprazolam 0.25 mg at bedtime, and they were instructed to fast as per standard ASA guidelines. Before administering study medications to the patient groups, patients were brought to the preoperative room the morning of surgery, where monitors were attached and baseline heart rate, systolic blood pressure, and diastolic blood pressure were measured. Patients in Group P got a placebo medicine and a sip of water 60 minutes before the anticipated anesthetic induction time, while those in Group C received oral clonidine in 200 microgram (μgm) doses. To constrict the nasal mucosal arteries, each patient got a nasal packing containing 2% xylocaine and 1:200000 adrenaline. After getting the study medications for 60 minutes, the patients were then taken to the operating room. During the pre-induction stage, the patients were all fitted with pulse oximetry, electrocardiography leads, and a non-invasive blood pressure monitor cuff. The findings for heart rate and blood pressure were then recorded. The 15-20 ml/kg Ringer's lactate solution was infused into the patient through an intravenous (IV) line with an 18G cannula. After the patients had been pre-oxygenated with 100% oxygen for three minutes, they were given fentanyl (2 μg/kg IV), glycopyrrolate (4 μg/kg IV), ondansetron (4 mg IV), and midazolam (2 mg IV). The patients were then given 0.1 mg/kg of the muscle relaxant vecuronium bromide after being given propofol (1%) IV until they lost vocal command (up to 3 mg/kg at most). Under direct laryngoscopic observation, endotracheal intubation was performed while extended 100% oxygen mask breathing lasted for three minutes. To keep the anesthesia going, sevoflurane 1.5%-2% and 66% nitrous oxide was used. Vecuronium IV injections were given in the usual top-up amounts as and when they were required.

The heart rate and non-invasive blood pressure (NIBP) were measured at the time of induction, one and five minutes after intubation, then every 10 minutes for the following 30 minutes, and lastly every 15 minutes for the remaining 120 minutes of the surgical procedure. To maintain the mean arterial blood pressure (MAP) within 20% of the baseline, rescue bolus doses of propofol (10 mg/bolus) were intended to be used to treat any intraoperative hypertension episodes. If that didn't work, a 2 μg/kg IV fentanyl bolus was to be administered. If the intended outcome was still not achieved after taking both medications, a continuous infusion of diluted nitroglycerin 100 μg/ml injection was to be administered as 0.5-10 μg/min infusion, and the condition was to be recorded. Similar to this, any incident of intraoperative hypotension was treated with increasing doses of mephentermine sulfate injection (3 mg IV) until normotensive pressures were reached. Any decrease in blood pressure greater than 20% from the initial number is referred to as hypotension. Corrective treatment was administered to patients in cases where such a fall was seen, as previously stated.

The operative surgeon graded the surgical area for bleeding using the six-point average category scale (Table 1) [1].

**Table 1:** Six-point average category scale

Finding	Score
No bleeding	0
Slight bleeding: no suctioning of blood required	1
Slight bleeding: occasional suctioning required; surgical field not threatened	2
Slight bleeding: frequent suctioning required; surgical field threatened by bleeding a few seconds after suction was removed	3
Moderate bleeding: frequent suctioning required; bleeding threatened the surgical field directly after suction was removed	4
Severe bleeding: constant suction required; bleeding appeared faster than could be removed by suction surgical field severely threatened and surgery was not possible	5

Surgery conditions two and three were set for the ideal category scale of values. If any instances of undesirable consequences were recorded, neostigmine and glycopyrrolate injections given in standard dosages were used to reverse anesthesia. Extubation was started when the patient responded to verbal orders, had a strong gag reflex when the oropharynx was stimulated with a suction catheter, and showed respiratory movement in the chest and reservoir bag. Atropine injections in titrated doses of 0.6 mg IV were to be given if the heart rate dropped by more than 20% of the baseline at any point during surgery. If the patients' MAP dropped by more than 20% of the baseline, IV fluid infusions and/or injections of ephedrine 6 mg IV in supplements were to be tried as a treatment.

Statistical methods

For normally distributed quantitative parameters, the mean values were compared between study groups using an independent sample t-test (two groups). A P value of more than 0.05 was considered statistically non-significant, a P value of 0.05 or less was considered statistically significant, and a P value of 0.001 was considered statistically highly significant. The study was carried out using IBM SPSS Statistics for Windows, Version 20.0 (Released 2011; IBM Corp., Armonk, New York, United States). We chose 60 patients, giving 200 μg of tab clonidine to 30 in Group C and a placebo to 30 in Group P 60 minutes before the scheduled operation. To prevent bias, the tablets were packaged in packages that looked identical.

## Results

As observed in Table 2, the demographic observations are as follows: the mean age in Group C was 44 ± 7.11 and in Group P was 43.6 ± 7.34 (P = 0.83) statistically non-significant. The gender-wise distribution of study participants among the two groups was statistically not significant (P = 0.5). The distribution of ASA physical status I and II among the study cases in both groups was statistically non-significant. The distribution of surgeries performed under FESS in both groups was found to be statistically non-significant (P >0.05). This depicts the demographic data to be statistically non-significant.

**Table 2:** Demographic distribution in study participants n: the number of patients from the respective group; NS: non-significant; SD: standard deviation; ASA: American Society of Anesthesiologists; DNS: deviated nasal septum

Parameter		Group		P-value
		Clonidine (n)	Placebo (n)	
Mean ± SD Age (years)		44 ± 7.11	43.6 ± 7.34	0.83 NS
Female		6	7	0.5 NS
		20.0%	23.3%	
Male		24	23	
		80.0%	76.7%	
ASA Class I		13	14	> 0.05 NS
		43.3%	46.6%	
ASA Class II		17	16	
		56.6%	53.3%	
Name of procedure	Nasal Polyp	5	5	> 0.05 NS
	DNS with bilateral Concha Bullosa	1	0	
	Sinusitis	24	25	

As observed in Table 3, the difference in heart rate (HR) was not significant at the time of recording the baseline value and the value at 120 min mark. The difference in heart rate was statistically significant at induction and one min, five min, 10 min, 20 min, 30 min, 45 min, 60 min, 90 min, and 105 min along the duration of the study.

**Table 3:** Comparison of HR among the study participants HR: heart rate; BPM: beats per minute; SD: standard deviation; S: significant; NS: non-significant

HR (bpm)	Group	Mean HR (bpm)	± SD	P-value
Base	Clonidine	75.97	9.57	0.271 NS
	Placebo	73.53	7.19	
Induction	Clonidine	89.67	13.23	0.001 S
	Placebo	101.37	12.75	
1 min	Clonidine	95.07	12.05	0.021 S
	Placebo	101.80	9.79	
5 min	Clonidine	97.83	9.96	< 0.001 S
	Placebo	106.63	8.11	
10 min	Clonidine	95.50	13.43	0.004 S
	Placebo	103.80	6.22	
20 min	Clonidine	90.87	9.65	0.001 S
	Placebo	98.17	5.08	
30 min	Clonidine	82.10	11.13	< 0.001 S
	Placebo	94.37	8.58	
45 min	Clonidine	79.93	11.37	0.005 S
	Placebo	86.63	4.93	
60 min	Clonidine	77.87	12.99	< 0.001 S
	Placebo	89.10	7.34	
75 min	Clonidine	100.57	7.84	0.016 S
	Placebo	106.97	11.68	
90 min	Clonidine	75.87	10.64	< 0.001 S
	Placebo	89.13	5.83	
105 min	Clonidine	76.17	10.65	< 0.001 S
	Placebo	87.60	5.39	
120 min	Clonidine	86.77	9.10	0.146 NS
	Placebo	90.67	11.27	

As observed in Table 4, the difference in MAP was not significant at the time of recording the baseline value and the value at 120 min mark. The difference in mean arterial pressure was statistically significant since induction and at one min, five min, 10 min, 20 min, 30 min, 45 min, 60 min, 90 min, and 105 min during the duration of the study.

**Table 4:** Comparison of MAP among the study participants MAP: mean arterial pressure; SD: standard deviation; S: significant; NS: non-significant; mmHg: millimeters of mercury

MAP (mmHg)	Group	Mean (mmHg)	± SD	P-value
Base	Clonidine	92.33	8.04	0.71 NS
	Placebo	91.70	4.62	
Induction	Clonidine	91.17	10.33	< 0.001 S
	Placebo	103.30	9.01	
1 min	Clonidine	96.40	7.19	< 0.001 S
	Placebo	115.07	8.03	
5 min	Clonidine	87.90	6.29	< 0.001 S
	Placebo	103.47	7.11	
10 min	Clonidine	69.40	7.57	< 0.001 S
	Placebo	95.10	7.07	
20 min	Clonidine	76.33	5.31	< 0.001 S
	Placebo	90.20	10.62	
30 min	Clonidine	66.57	7.28	< 0.001 S
	Placebo	92.73	5.84	
45 min	Clonidine	71.03	6.37	< 0.001 S
	Placebo	92.30	6.53	
60 min	Clonidine	69.43	5.27	< 0.001 S
	Placebo	81.27	5.83	
75 min	Clonidine	75.63	6.77	< 0.001 S
	Placebo	82.33	5.11	
90 min	Clonidine	75.90	9.30	< 0.001 S
	Placebo	84.97	7.25	
105 min	Clonidine	81.60	9.14	0.016 S
	Placebo	86.70	6.42	
120 min	Clonidine	81.97	9.82	0.097 NS
	Placebo	85.83	7.79	

As observed in Table 5, the blood loss grading wise distribution of study participants among the two groups was significant (P < 0.001). This difference was statistically significant.

**Table 5:** Blood loss grading wise distribution of study participants among the groups n: the number of patients from the respective group; S: significant

Blood Loss Grading	Group		P-value
	Clonidine (n)	Placebo (n)	
2	13	0	< 0.001 S
	43.3%	0.0%	
3	15	10	
	50.0%	33.3%	
4	2	20	
	6.7%	66.7%	
Total	30	30	
	100.0%	100.0%	

The mean duration of surgery in Group C was 145.07 ± 15.17, and in Group P it was 164.9 ± 11.69 (P < 0.001). This difference was statistically significant.

Clonidine benefits as a premedicant are that it induces sedation and anxiolysis and reduces the stress reaction to intubation and laryngoscopy. By regulating heart rate and blood pressure, it maintains intraoperative cardiovascular stability, maintains normal respiratory function, results in fewer upsetting side effects like nausea and vomiting, and effectively provides analgesia to the patient during and after surgery. It is also simple to administer. Patients between the weights of 40 to 65 kg take clonidine well at a dose of 200 μgm without experiencing any significant side effects. In the FESS, premedication with oral clonidine significantly enabled controlled hypotension and a clear surgical field, which was helpful for a successful operation with a successful anesthetic outcome.

## Discussion

**Figure 1:**
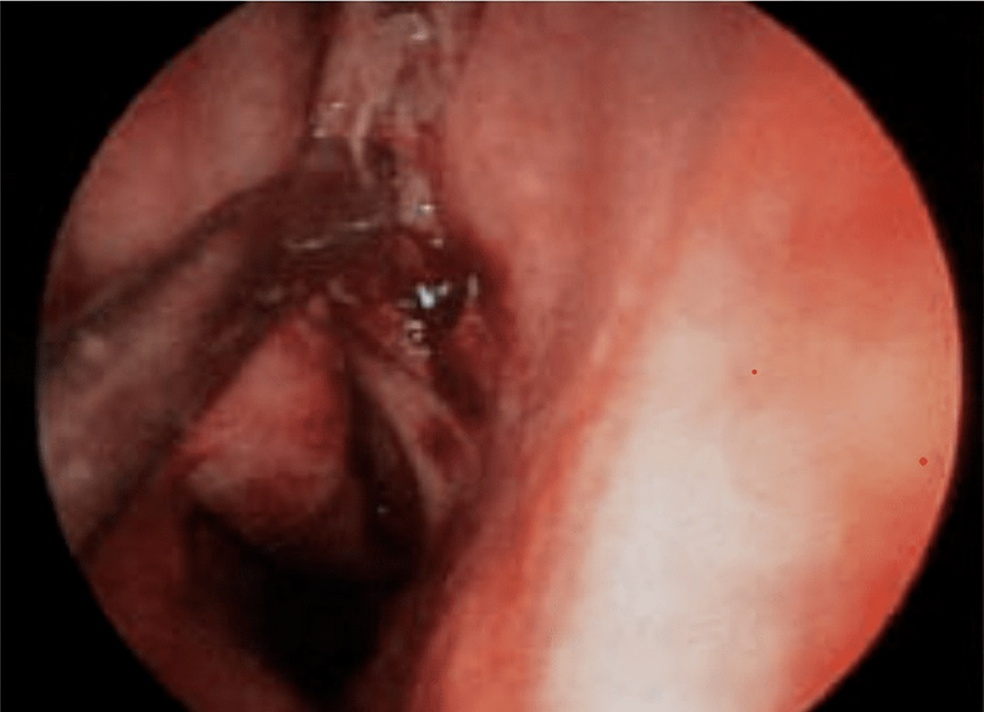
Endoscopic view of instrumentation of nasal septum Image captured during FESS at the place of study. FESS: functional endoscopic sinus surgery

For the treatment of chronic rhinosinusitis that is unresponsive to medical intervention, FESS is recommended. One of the most common matters of concern in endoscopic sinus surgery is the control of bleeding because the nasal sinus mucosa has a rich vascular supply [2]. In FESS, the highest risk is related to capillary bleeding and capillary circulation, which can be decreased by controlling the arterial blood pressure, causing constriction of blood vessels [3]. Bleeding could affect the surgical site as well as the patient’s body temperature [4] and obscure the clarity of the site of surgery, causing a longer time for the procedure to accomplish [5] (Figure 1).

Our aim in FESS is to lower systemic vascular resistance or heart output [6]. There are some recommendations for medications and anesthetics to lower blood pressure and reduce hemorrhage [6-8]. Numerous vasoconstrictor drugs, such as phenylephrine hydrogen chloride (HCl) (0.25%), sodium nitroprusside, esmolol, oxymetazoline HCl (0.05%), cocaine (4%) [9], and isoflurane (1% by inhalation) [10] have been investigated as the drugs for controlling hypotension [1]. The requirement and technique of controlled hypotension were thought to provide a bloodless surgical field and reduce blood loss, particularly during FESS or any other surgical procedure where the surgeon's ability to have a vision of the surgical area can be adversely affected by even a small amount of bleeding. Controlled hypotension is known to raise the risk of ischemia of vital organs, and intraoperative bleeding may not always be related to blood pressure, in addition to the possibility that hypotension alone may not improve the surgical field [11]. As a centrally active antihypertensive and α2-adrenergic agonist, clonidine has been shown in studies to be able to stabilize the circulatory system and reduce stress response during the perioperative period [12]. Clonidine affects the neurons that receive baroreceptor afferents, which reduces sympathetic outflow while increasing centrally derived parasympathetic tone and lowering heart rate [13]. It also changes tonic and phasic blood pressure regulation, causing a drop in blood pressure because it is an α2-adrenoceptor agonist. To improve the surgical field's quality, a decrease in bleeding is needed, which can be achieved by clonidine's ability to reduce blood pressure and heart rate. Clonidine affects the strength of postjunctional α1-adrenoceptor-mediated vasoconstriction, though the exact mechanism is still unknown [12]. To decrease bleeding during FESS and stabilize the patient's intraoperative hemodynamic condition, clonidine can constrict peripheral blood vessels, lower systemic blood pressure, and slow the heart rate [14]. Clonidine can also be used as a premedicant to treat hypotension, lessen the need for intravenous and volatile anesthetics, and block the pressor reaction during intubation.

In our study, we found that the demographic observation in Table 2 was as follows: in age, the mean in Group C was 44 ± 7.11, and in Group P was 43.6 ± 7.34 (P=0.83). The difference was statistically non-significant among the groups. In the gender distribution, the percentage of males was 80.0% vs. 76.7% and the percentage of females was 20.0% vs. 23.3% in Groups C and P, respectively (P=0.5). The difference was statistically non-significant among the groups. The classification was 43.3% vs. 46.6% in ASA I and 56.6% vs. 53.3% in ASA II for the study Groups C and P respectively, and was found to be statistically non-significant (p >0.05). The distribution of surgeries performed under FESS was as follows: sinusitis (24 in number), nasal polyps (five in number), deviated nasal septum (DNS) with bilateral concha bullosa (one in number) in Group C, and sinusitis (25 in number) and nasal polyps (five in number) in Group P. The difference was found to be statistically non-significant (P >0.05). When it comes to heart rate, our research found that there was no significant difference between the heart rates of the clonidine-premedicated Group C and the placebo-premedicated Group P upon entering the operating room (Table 3), supporting the results of a prior study [15]. Our study also shows that there is a significant difference in heart rate between Group C and Group P (Table 3), beginning with the induction and continuing for one minute, five minutes, 10 minutes, 20 minutes, 30 minutes, 45 minutes, 60 minutes, 75 minutes, 90 minutes, and 105 minutes, which was the time when the majority of the surgical intervention was taking place. However, at the 120th minute, the variation in heart rate became insignificant. In our research, neither the placebo Group P nor the clonidine Group C had bradycardia. In prior research [16] that examined the relationship between HR and the grade of surgical field bleeding, it was discovered that a lower HR was associated with less surgical field bleeding. In our research, the surgical field quality was unaffected because there was no decrease in HR during any of the observational periods.

It has been hypothesized that during general anesthesia, a decrease in MAP can reduce intraoperative bleeding [17,18]. However, venous pressure and capillary blood flow are also taken into consideration alongside the MAP when it comes to hemodynamic variables and the extent of surgical bleeding, even though, according to published studies [16], total blood loss and MAP do not necessarily have to be correlated. ꞵ blockers are known to lower myocardial contractility, slow the heart rate, and indirectly affect MAP. Additionally, the vasoconstriction of the mucosal membrane of arterioles and precapillary sphincters brought on by the unopposed adrenergic actions of endogenous catecholamines may be responsible for the enhanced surgical field [19]. The mean arterial blood pressure was significantly lower in our clonidine Group C in comparison to Group P (Table 4) since induction till 105 min found at one min, five mins, 10 mins, 20 mins, 30 mins, 45 mins, 60 mins, 75 mins, 90 mins, 105 mins of induction, though the difference of MAP found to be statistically non-significant at 120 mins. In our study, we observed episodes of hypotension (fall of more than 20% from baseline) at the 10 min, 30 min, 45 min, and 60 min marks (MAP) in a few patients in Group C for which corrective treatment of injection mephentermine sulfate 3 mg IV was given and recorded. This is evidenced by the normotensive findings observed at the 20 min, 40 min, 55 min, and 75 min marks respectively, which is concurrent with the findings of a previous study [15].

A previous study [20] found that prostaglandin E1 and clonidine both decrease blood loss during paranasal sinus surgery without resulting in hypotension. They found that the reduced blood loss was due to clonidine's capacity to constrict peripheral blood vessels and lessen blood flow to the nasal mucous membrane. We discovered that the clonidine group had substantially more patients with better (lower) surgical grades than the placebo group did, which led to higher surgeon satisfaction. These findings are consistent with those of earlier research [21]. There were fewer hemorrhages in the clonidine group. (Table 5). In the patients of study groups C and P, the category scale score for the evaluation of the intraoperative surgical field grade 2 was found to be 43.3% vs. 0.0%, grade 3 vs. 33.3%, and grade 4 vs. 66.7%. Statistics showed that there was a significant variation between the groups (P < 0.001). No patients in either group experienced blood loss that necessitated surgical or postoperative blood transfusions. Although we kept track of the entire time the surgeries were performed, our research period lasted for 120 minutes after induction.

We discovered that the placebo group had greater bleeding severity scores of 3 and 4 than the clonidine group (Table 5) [10,22]. Higher doses may cause α-stimulation, which could raise blood pressure [15], although a previous study stated that 4 μg/kg has typically been used in human studies without any signs of α-stimulation. We chose oral clonidine 200 μg for premedication in our study group because it significantly reduced anxiety compared to 100 μg and did not result in hypotension that persisted in the postoperative period as it did with 300 μg. The same result was also supported by prior research [23]. The inefficiency of having an accurate dosage while scoring a drug in a tablet preparation into smaller fragments was the reason for not using the drug dose as per kilogram of body weight. Therefore, in the current research, we gave all of the patients in our study groups a fixed dose of 200 μg of clonidine orally 60 min before induction. Patients who got oral clonidine as a premedicant were shown to be at ease before the surgery, in contrast to the placebo group patients who were found to be anxious about the procedure. It was intended to use rescue bolus doses of propofol (10 mg/bolus) to treat any intraoperative hypertensive episode The rescue medication was not needed because there was no hypertensive crisis seen. All of the patients in the clonidine group, as well as a few others who were asleep but alert endured extubation without any problems. The patients in the placebo group who were administered inhalational anesthetics for a longer period of time required prolonged observation during extubation and also in the postoperative period. In the study instances, bradycardia, nausea, and dizziness-side effects of clonidine-were not reported to cause complications.

Limitations

Studies in ASA physiological status beyond class II were not undertaken during our observation. No comparison with other hypotensive agents was considered. The requirement for the quantity of anesthetic agents used was not addressed in our study. Since clonidine is prone to exacerbate cardiovascular status (ASA Class III), there are some restrictions on its use in individuals with bradycardia, conduction abnormalities, and cardiovascular instability. In patients with airway obstruction, obesity, or at extremes of age, it is best to avoid or should be taken with caution due to the possibility of unwanted action.

Scope

Studies can be conducted to learn about the analgesic and sedative effects of the drug clonidine during intraoperative and postoperative periods of FESS.

## Conclusions

The current prospective randomized placebo-controlled double-blind study demonstrates that oral clonidine premedication in a dose of 200 μg 60 minutes before induction of anesthesia in functional endoscopic sinus surgery (FESS) under general anesthesia is effective in achieving controlled hypotension. Additionally, it reduces (almost negligibly) the need for extra hypotensive drugs to clear the operating field, providing a significant reduction in bleeding as well as better surgical field quality, as demonstrated by a decreased need for intraoperative suctioning. The drug neither created oversedation nor caused significant hypotension or bradycardia, which would have extended the recovery time. So, we can claim that oral clonidine can be given during FESS as a premedicant and a hypotensive agent 60 minutes before the induction of anesthesia.
